# Dispersal and spatial heterogeneity allow coexistence between enemies and protective mutualists

**DOI:** 10.1002/ece3.1151

**Published:** 2014-09-18

**Authors:** Timothée Poisot, James D Bever, Peter H Thrall, Michael E Hochberg

**Affiliations:** 1 Université Montpellier II, Institut des Sciences de l'Evolution, UMR 5554Place Eugène Bataillon, 34095, Montpellier, CEDEX 05, France; 2 Département de Biologie, Université du Québec à Rimouski300 Allée des Ursulines, Rimouski, Quebec, G5L 3A1, Canada; 3 Québec Centre for Biodiversity SciencesMontréal (QC), Canada; 4 Department of Biology, Indiana UniversityBloomington, Indiana, 47405; 5 CSIRO Plant IndustryGPO Box 1600, Canberra, Australian Capital Territory, 2601, Australia; 6 Santa Fe InstituteSanta Fe, New Mexico, 87501; 7 Wissenschaftskolleg zu BerlinBerlin, 14193, Germany; 8 School of Biological Sciences, University of CanterburyPrivate Bag, 4800, Christchurch, 8140, New Zealand

**Keywords:** Community ecology, source-sink dynamics, metacommunities, host-symbiont interactions, coexistence theory, spatial dynamics

## Abstract

Protective mutualisms, where a symbiont reduces the negative effects of another species on a shared host, represent a common type of species interaction in natural communities, yet it is still unclear what ecological conditions might favor their emergence. Studies suggest that the initial evolution of protective mutualists might involve closely related pathogenic variants with similar life histories, but different competitive abilities and impacts on host fitness. We derive a model to evaluate this hypothesis and show that, in general, a protective variant cannot spread from rarity or exclude a more pathogenic strain. While the conditions allowing mutualist invasion are more likely with increased environmental productivity, they still depend on initial densities in the invaded patch exceeding a threshold, highlighting the likely importance of spatial structure and demographic stochasticity. Using a numerical simulation approach, we show that regional coexistence is in fact possible in an explicitly spatial system and that, under some circumstances, the mutualist population can exclude the enemy. More broadly, the establishment of protective mutualists may be favored when there are other life-history differences from more pathogenic symbionts, such as vertical transmission or additional direct benefits to hosts.

## Introduction

Species interactions are fundamental drivers of ecological and evolutionary dynamics in natural ecosystems. Consequently, there is a vast literature on the role of predator–prey and competitive interactions in structuring communities (Fussmann et al. 2007) and increasing attention given to parasites, pathogens, and herbivores (Cornell et al. 1998; Lafferty et al. 2006; Thrall et al. 2007). However, despite considerable work on the evolutionary ecology of mutualisms (Bronstein 1994; Leigh and Rowell 1995; Herre et al. 1999; Moran and Wernegreen 2000; Moran 2007; Léotard et al. 2008), relatively little is known about how environmental conditions affect their persistence or community impacts (Hochberg and van Baalen 2000; Hochberg et al. 2000; Thrall et al. 2007). This is surprising given that a significant proportion of species interactions involves some form of mutualism [e.g., approximately 80% of land plants form associations with mycorrhizal fungi (Smith and Read 1997)] and is thus likely to have pervasive roles in evolutionary and ecological processes.

A limited set of environmental variables is thought to determine the stability and diversity of mutualistic interactions (Bronstein et al. 2003, 2006; Thrall et al. 2007; Jones et al. 2009). In particular, energy flow through parasite communities is considered an important driver of species diversity (Poulin 2010), and theory indicates that it should influence, more generally, the structure and function of symbiotic communities (we use symbiosis to mean any persistent biological interaction between individuals of two species) (Thrall et al. 2007). Most evidence for this comes from theoretical treatments of “providing” mutualisms in which one species benefits another by providing or facilitating access to otherwise rare or missing resources, resulting in increased growth or reproduction. A central prediction is that increasing environmental productivity should result in a shift toward symbiont antagonism and virulence in terms of types of species interactions and individual effects, respectively (Hochberg et al. 2000; Neuhauser and Fargione 2004; Thrall et al. 2007).

Protective mutualisms, defined as those in which one symbiont reduces the negative effects of another symbiont or of a natural enemy such as a parasite or predator on a shared host, are canonical examples of trophic interaction modifiers, which can have pervasive effects on the organization and evolution of host–symbiont communities (Golubski and Abrams 2011). These include the stimulation of host defenses (Talham et al. 1999; Bennett et al. 2009), impacts on pathogen virulence or life cycles, competitive exclusion (Blanco et al. 2001), or intraguild antagonism (Rosenheim et al. 1995; Holt and Hochberg 1998). These effects all result in the reduction of host–antagonist contacts or associated negative impacts. The most dramatic examples include invasion facilitation (Clay and Holah 1999; Clay et al. 2005), changes in landscape cover (Goheen and Palmer 2010), or more complex imbrications of symbioses through interactions with multiple mutualists (Scott et al. 2008).

How protective mutualists arise and persist is still an open question. Theoretically, they could originate from pathogenic associations (Genkai-Kato and Yamamura 1999), although phylogenetic analyses question this view (Moran and Wernegreen 2000; Sachs and Simms 2006). They may also differ from “providing mutualists” (e.g., rhizobial bacteria) with regard to environmental features that promote their emergence. Expected occurrence of protective mutualisms in relation to environmental factors (e.g., maximum growth rate of host populations, hereafter called “productivity”) has not been well explored. Theory suggests that such symbioses are likely to increase in frequency with greater environmental productivity and corresponding increases in the prevalence of antagonists (Hochberg and van Baalen 2000).

We develop a theoretical model to investigate how environmental and demographic parameters affect the relative abundance of enemies (predators, pathogens, or parasites) and “conditional protective mutualists” interacting with a host population (e.g., Nuismer et al. 2003). Conditional protective mutualists (hereafter called “mutualists”) have context-dependent effects, as they both exploit the host population and protect it from exploitation by enemies (e.g., Chamberlain & Holland 2009). We assume that the mutualist and enemy share a common evolutionary origin, with one potentially originating through mutation in a resident population of the other, or through spatial isolation, local adaptation, and subsequent mixing; under this scenario, it is reasonable to expect that mutualists and enemies have similar life histories and trait values. We focus on situations where such mutualists protect hosts through direct exploiter removal and investigate their potential to invade existing host–enemy interactions. We assume that enemies and mutualists are obligate biotrophs, but do not consider recovery from infection, multiple infections, or host immunity. Despite these simplifications, our model employs general features of host–symbiont associations (Anderson and May 1981; Hochberg and Holt 1990; Genkai-Kato and Yamamura 1999), including infection, virulence, birth and mortality rates, and the potential for host regulation by the enemy or by the mutualist in the absence of the enemy.

## Model development

We model a host–enemy–mutualist association as follows. We assume that the enemy (whose density is noted as *E*) and the mutualist (*M*) have direct negative effects on the host (*H*) and that the mutualist's positive effect is due to the protection it provides by lowering the enemy attack rate and by having lower effects on mortality compared to the enemy, or through a combination of both. This may result in fewer hosts attacked and fewer enemies produced. Thus, the net effect of such a mutualist is a balance between its detrimental and protective effects (i.e., mutualistic effects are contingent on enemy abundance). Our model is structurally similar to earlier models of directly competing species which exploit a dynamic resource base (Hochberg and Holt 1990; Holt and Hochberg 1998; Gyllenberg et al. 2006) with the notable exception that here the mutualist reduces competitor abundance without directly increasing its own population. We assume a linear numerical response of both the enemy and the mutualist to host density. Instantaneous changes in host, enemy, and mutualist population densities are given by:
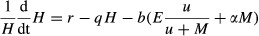
1a
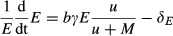
1b
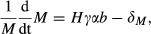
1cwhere *r* is the intrinsic host growth rate, assumed to correlate with environmental productivity (Leibold 1989), and *q* the crowding factor regulating the strength of density-dependent mortality. For simplicity, progeny size (*γ*) and the transmission parameter (*b*) are assumed equal for both organisms, as might occur during early stages of evolutionary divergence. Mutualists can be either more (*α* > 1) or less (*α* < 1) virulent than the enemy with regard to impacts on host mortality (we assume *α* = 1 for the enemy, thereby simplifying eq. 1a-c). Note that virulence is defined as the direct per capita impact of the symbiont on host mortality and that virulence of the mutualist affects its population growth rate directly; this is because we assume that symbiont growth is linearly related to symbiont impact on the host, and therefore, a reduction in host mortality comes at the cost of slower growth with regard to more virulent strains. There are a variety of biological situations in which this assumption holds, including organisms that require victim mortality to transmit or spread, such as parasitoid wasps and bacteriophages (Eggleton & Gaston 1990), micropredators and grazers, which grow by consuming biomass from the victim population, and host–parasite systems in which host condition deteriorates as parasite density increases (Beldomenico and Begon 2010). In all of these systems, lower impact on host mortality is linked to slower growth. Finally, *δ*_*M*_ and *δ*_*E*_ are the intrinsic mortality rates of mutualist and enemy, respectively, and *u* (assumed to be greater than 0) describes the effect of the mutualist on the enemy (the lower *u*, the more the mutualist is able to defend the host).

When the enemy is present, the mutualist has both negative and positive effects on the host. We define a criterion for net mutualism (*U*) based on differences between equilibrium host population size in the presence of the mutualist alone (H^*^_M_) versus the enemy alone (H^*^_E_).
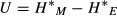
4

The mutualist provides a net benefit to the host when *U* > 0. The two nontrivial host equilibria when either mutualist or enemy is present are given, respectively, by:
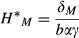
3a
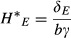
3b

From equations 3 and (1) a mutualist will have a net protective effect if
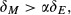
7that is, when the mutualist enjoys little gain from host exploitation (low *α*), and the mutualist per capita death rate is high relative to that of the enemy.

## Results

### Condition for pathogen invasion in a mutualist–host interaction

We analyze the condition for invasion of a pathogenic mutant into a mutualist–host interaction. The two species equilibrium for hosts is H^*^_M_ (3a) and for mutualists is 
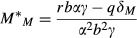
8

This equilibrium yields positive values assuming that *rbαγ* > *qδ*_*M*_, as we do for the remainder of the study. Note that the situations in which the enemy cannot invade a resident mutualist correspond to sufficiently high values of the virulence parameter *α* such that the mutualist is actually more virulent than the enemy. Assuming that the system is at this equilibrium, the enemy mutant can invade from rarity *(*i.e., d*E/*d*t *> 0) when
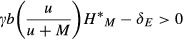
9

By replacing (3a) and (5) into inequality (6) and with parameter values that satisfy (4), we find that the enemy can invade the resident mutualist if
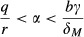
10

This condition implies that the pathogen can invade the resident mutualist if *r* (environmental productivity) is sufficiently large, and host self-limitation is sufficiently low.

### Condition for mutualist invasion into a host–enemy interaction

We first derive the condition for initial invasion of the mutualist into a resident population of the host and its enemy at equilibrium. In the absence of the mutualist, the system has a single nontrivial equilibrium, which is always stable to small perturbations (Appendix S1). The host equilibrium (H^*^_E_) is given in equation (3b), and the associated equilibrium enemy density (E^*^) is
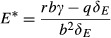
11

The condition for mutualist invasion from rarity is found by evaluating equation (1c) when the mutualist population approaches zero and the host and enemy are at the equilibrium densities given by equations (3a, 5), or
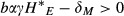
12which admits a single solution
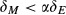
13

No solution simultaneously satisfies inequalities (10) and (4), indicating that for the mutualist to invade; its net effect *U* must be negative (i.e., it may have a partial positive effect by decreasing enemy abundance, but its overall effect on the host is antagonistic). However, numerical experiments reveal that when (4) is satisfied, the mutualist may invade when its initial density is above a threshold (Amarasekare 1998, 2004; Morales et al. 2007). To find the critical value of the mutualist population density above which it can invade (m*), we substitute *m* for *M* in eqn (1a-b) and solve at the equilibrium to yield
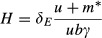
14

The criterion for mutualist invasion (m = m*) is found by substituting (11) into inequality (12), or
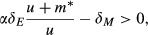
15which is true when
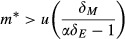
16

**Figure 1:**
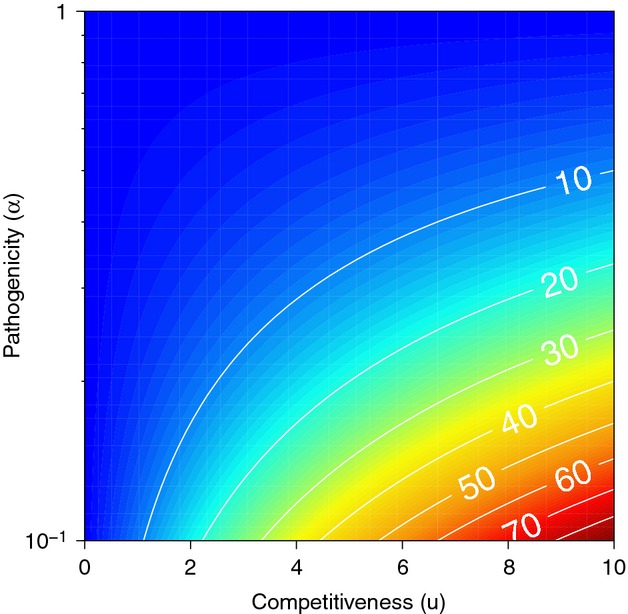
Minimal number of mutualists m* required to invade a resident system of the host and enemy at equilibrium, under the hypothesis that mutualists are introduced through immigration (eq. 14). Several model parameters influencing mutualist and enemy populations are important in determining m*. Mutualist invasion is facilitated when they tend to harm their host (high *α*) and impact the resident enemy (low values of *u*). The consequences of this result are discussed in the main text. Parameter values for this figure (*δ*_*E*_ = *δ*_*M*_) were chosen to ensure that eq. 2 holds (i.e., net mutualism) for any *α* < 1.

If the mutualism criterion is satisfied (i.e., *U* > 0), then the right hand side of (13) is always positive. The population size required for invasion decreases when the virulence of mutualists is reduced (low *α*), or when mutualists have a strong protective effect (low *u*) (Fig.1). Should the mutualist replace the resident enemy, the system will return to the stable host–mutualist equilibrium shown in Appendix S1. Condition (13) may be satisfied in at least two ways: (1) through persistence on another host species in the local community and sufficient cross-host transmission to also persist on the focal host (Redman et al. 2001; Goodrich-Blair and Clarke 2007) or (2) when the mutualist is produced by hosts in one or more neighboring patches, and the former's density on local hosts is augmented via immigration (Palmer et al. 2003; Thompson 2005). Below we investigate the latter case.

### Invasion of the mutualist through migration from a neighboring patch

To better understand the conditions under which a mutualist may invade a resident enemy population through immigration, we analyze a modified version of the model with two patches, one with the mutualist and the host (patch 1) and the other with the enemy and the host (patch 2). Such a scenario is not unrealistic; empirical studies have demonstrated geographical structure in symbiont populations, with (genetically) different strains of the same species shown to have either antagonistic or mutualistic effects on the same host depending on location and local environment (Thompson and Fernandez 2006). The population dynamics in the receiving patch are given by equation (1a-c), and population dynamics in the source patch (1) are given by
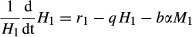
14a
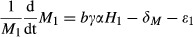
14b

**Figure 2:**
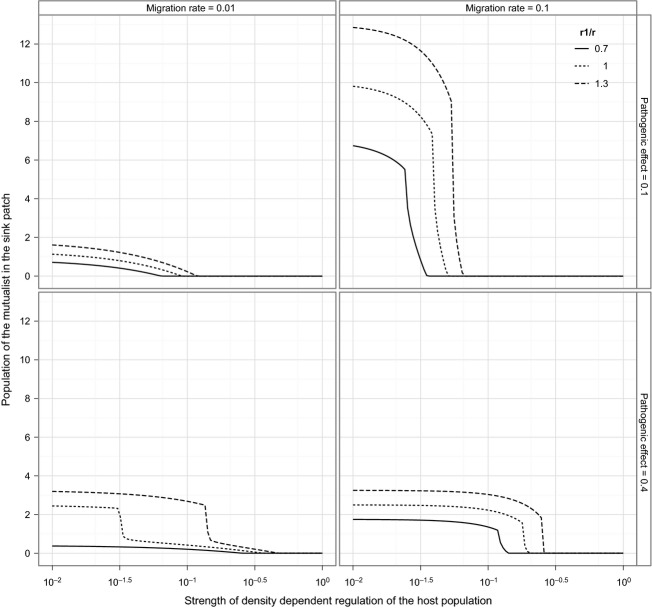
Mutualist population size in the sink patch of the two-patch model. The invasion and maintenance of mutualists in the sink is favored by increasing host productivity in the source patch (*r*_1_), the migration rate (*ε*_1_), and decreasing the strength of density-dependent regulation of the host population (*q*). Simulations were initiated with no mutualists in the sink patch. Population size was measured as the mean of the last 200 generations of a simulation running for 2000 generations. Parameters: *b* = 1, (*δ*_*E*_ = *δ*_*M*_) = 0.1, *r* = 1, *γ* = 0.1, *u *=* *1. Although not shown here, low values of *u* resulted in more mutualists persisting in the sink patch.

We assume that productivity (*r*,*r*_1_) differs between patches so as to reflect variation in habitat quality, but that density-dependence (*q*) is intrinsic to the host population (e.g., through intraspecific competition). We also assume constant directional emigration from patch 1 to patch 2, at rate *ε*_1_. The dynamics in patch 2 are given by equation (1a-c), where (1c) is modified to receive an additional *ε*_1_ *M*_1_ to account for immigration. Numerical simulations of this system were conducted by varying *q*,*r*_1_*/r*,*u*, and *ε*_1_ and investigating their effects on the ability of mutualists to invade and establish (Fig.2). We observe that high values of *q* protect the resident enemy, even at high migration rates. In the two-patch system, with intermediate values of the migration rate and density-dependence, the two symbionts can coexist on the same host.

### Regional coexistence of symbiont types in a spatially explicit system

In the previous sections, we show that invasion of a conditional protective symbiont from rarity is not possible within a single population, but can occur in a two-patch system given a critical number of invading individuals, which is determined by the between-patch productivity gradient. We thus posit that in a broader context, assuming spatial variability in primary productivity (i.e., the intrinsic growth rate of hosts), regional coexistence between conditional protective mutualists and enemies is possible. To examine this prediction, we simulate the system on a square toric lattice of 10 × 10 patches, with a uniform dispersal kernel from one focal patch to all of its eight neighbors. Computer code (C++11) to reproduce the simulation can be found at https://github.com/tpoisot/ProtectiveMutualismModel (requires the *Gnu Scientific Library*).

We simulate the system by varying the dispersal rate of conditional protective mutualists and enemies, the pathogenic effect of protective mutualists, and primary productivity across patches. The primary productivity of each patch is drawn at random at the beginning of each simulation from a Gaussian distribution with mean *r* (the same as in the previous analyses of the model) and variance *v* (our measure of environmental heterogeneity). Based on results from the previous sections, we expect that the potential for regional coexistence between conditional protective mutualists and enemies will be greater in more heterogeneous landscapes. To measure regional coexistence, we record the proportion of mutualists in the total symbiont population (averaged over the last 1000 time-steps for a given set of input conditions). Values close to 0 or 1 indicate that enemies or mutualists dominate, respectively, whereas intermediate values signify coexistence.

**Figure 3:**
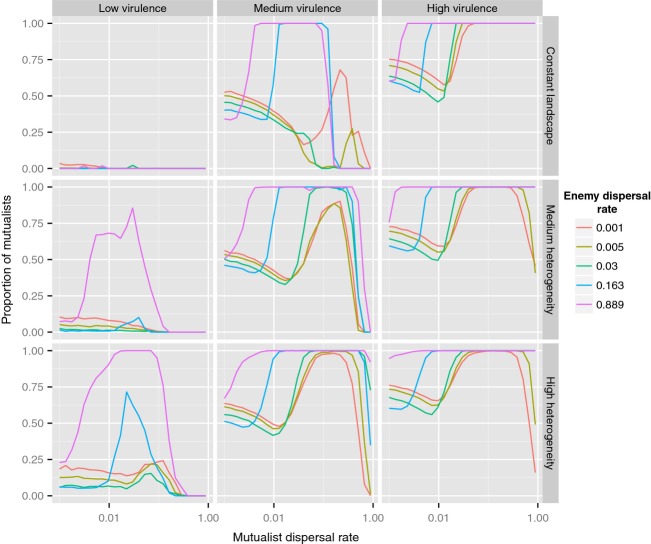
Proportion of mutualists over the whole landscape in the spatially explicit model. Relative virulence (*α*) varied in {0.1, 0.2, 0.3}, and the amount of heterogeneity in the distribution of patches growth rate (see main text) varied in {0, 0.5, 1}. As predicted in the two-patch analytical model, increased heterogeneity and dispersal rates allow the stable persistence of both mutualists and enemies, in some cases leading to the exclusion of enemies at the global scale.

As shown in Fig.3, the proportion of mutualists increases for pronounced negative effects of the mutualist (*α*) and highly heterogeneous environments. Mutualists are able to exclude the enemy for intermediate dispersal values, especially when the enemy disperses more than the mutualist. However, for most of the parameter combinations explored, spatial structure allows regional coexistence of both symbiont types. In full agreement with the predictions made above for the two-patch model, the only situation in which the enemy can drive the mutualist to regional extinction is in the absence of environmental heterogeneity. Finally, mutualists are only able to persist regionally for intermediate values of dispersal. When values of mutualism dispersal are too low, this prevents the establishment of source-sink dynamics as determined above for the two-patch system. Conversely, when dispersal is too high, mutualists are unable to sustain a local population and are eventually outcompeted by enemies.

## Discussion

Previous modeling work has shown that in systems of two enemies and one host, coexistence involving all three species is only possible if the more competitive enemy (analogous to the mutualist in the present study) also has a larger impact on host mortality (Hochberg and Holt 1990; Holt and Hochberg 1998). Our study extends this finding to show that coexistence based on reciprocal invasion from rarity is never possible when one of the enemies has a net mutualistic effect. Our results also highlight the central role played by environmental productivity (reflected by host population growth rate, *r*) in determining both the threshold for mutualist invasion and the production of migrating mutualists from alternative hosts or host populations in other patches. Host populations with higher *r* or lower intraspecific competition (*q*) are more likely to support the establishment and eventual maintenance of the mutualist in both the two-patch and spatially explicit systems. The small range of conditions resulting in the establishment of protective mutualists is in sharp contrast to the frequent observation, ecological importance, and broad taxonomic range of protective mutualisms in nature (Douglas 1994). Potential scenarios that can explain this discrepancy are (1) that protective mutualists and enemies arise from different phylogenetic and functional groups encompassing variation in relevant life-history traits, (2) that protective mutualists provide other types of benefits, and (3) that protective mutualists arise within spatially structured, environmentally heterogeneous environments. We discuss these alternatives below.

### Protective mutualists are not closely phylogenetically or functionally similar to the organisms from which they protect their host

The major conclusion of our mean field model is that invasion and persistence of a protective mutualist variant with otherwise similar biology to antagonists is not possible within a single homogeneous host population. This result suggests that protective mutualists arise from phylogenetically and functionally different groups of organisms from which they protect their host. While in some systems mutualists and antagonists represent sister species (Kawakita et al. 2010), phylogenetic studies of endosymbionts (Moran and Wernegreen 2000) suggest that symbionts do not necessarily originate from pathogens. In fact, many classic interactions involve enemies and protective mutualists that are not closely related, such as ant–plant–aphid interactions (Stadler and Dixon 2005). We suggest that invasion and spread of a protective mutualist are most likely for organisms that differ qualitatively in life histories (e.g., in host fitness effects, presence of free-living stages, transmission mode). For example, for many protective mutualists such as fungal endophytes, beneficial symbionts are vertically transmitted to offspring. Empirical studies on the emergence of mutualism in parasites of amoeba (Jeon 1972) or *Daphnia* (Ebert and Weisser 1997) show that vertical transmission favors reduced parasite virulence as predicted by theory (e.g., Fine 1975).

### Protective mutualists provide other types of benefits

Alternatively, protective mutualists may provide other benefits that increase the productivity of their hosts, thereby enhancing their probability of establishment and persistence. The distinction between protective and providing (i.e., increasing host growth or survival) mutualisms is not always straightforward to demonstrate empirically. Most symbiotic associations cannot be categorized as purely antagonist or mutualistic (Leung and Poulin 2008). While the spread of a protective mutualism is enhanced by covariation with higher host productivity, clearly this could be enhanced by other benefits to its host besides protection. In fact, plant fungal endophytes have strong negative effects on plant herbivores, but can also increase drought tolerance, phosphorus uptake, and competitive ability (Clay 1988). Moreover, classic nutritional mutualists (e.g., mycorrhizal fungi, rhizobia) can also enhance host defense against pathogens (Bennett et al. 2009). Thus, distinctions between nutritional and protective mutualists may be misleading – protection and providing could evolve jointly. Future modeling studies should consider this possibility although clearly such situations are more complex than the ones we model here. Another situation that can arise is the emergence of cheating strains in multistrain infections. Several studies (Harrison et al. 2006; Barrett et al. 2011) report that the emergence of such strains requires only a few mutations. As these strains are likely to be more competitive in the diseased environment, their spread can decrease virulence overall (Platt et al. 2012). This would represent a situation of conditional protective mutualism, in which the cheating strain directly decreases the relative fitness of the virulent strain. Future work should address how different mechanisms for conditional protection, or how covariation of protection with providing, might facilitate or restrict the emergence of mutualists.

### Protective mutualists arise within spatially structured, environmentally heterogeneous environments

Heterogeneity arising from asynchronous host dynamics in different patches could be sufficient to maintain mutualist–enemy coexistence. For example, plant fungal endophytes have strong negative effects on plant herbivores, but can also increase drought tolerance, phosphorus uptake, and competitive ability (Clay 1988). Moreover, classic nutritional mutualists (e.g., mycorrhizal fungi, rhizobia) can also enhance host defenses against pathogens (Bennett et al. 2009). Thus, as alluded to in the previous section, distinctions between providing and protective mutualists may sometimes be misleading – protection and providing could evolve jointly. Future modeling studies should consider this possibility, although clearly such scenarios are more complex than the ones we model here. Another situation that can arise is the emergence of cheating strains in multistrain infections. Several studies on bacteria report that the emergence of such strains may require only a few mutations (Harrison et al. 2006; Barrett et al. 2011). Because cheater strains do not bear the full costs of virulence, they can be more competitive for available resources in the disease environment, especially at low host density and thus competitively suppress more virulent genotypes (Platt et al. 2012). The cheater would then represent a conditional protective mutualist to the host.

Our analysis takes an ecological invasion approach involving three species to identify some of the main drivers of coexistence between protective mutualists and nonprotective enemies. Future studies should consider how host–enemy coevolution, the evolution of enemy transmission pathways, and more realistic environmental contexts (e.g., community composition, spatial structure, etc.) affect the strength and direction of these associations.

## Conflict of Interest

None declared.

## Supporting Information

**Figure S1** Stability of the Host-Enemy equilibrium. **Figure S2** Population size of the host (blue) and enemy (red) populations with sets of parameters predicting either neutrally stable (top caption) or oscillating (bottom caption) equilibrium (see Appendix S1). **Figure S3** Stability of the Host-Mutualist equilibrium.

Appendix S1 Stability of the two-species equilibria
