# Supplementary material for: Dispersal and spatial heterogeneity allow coexistence between enemies and protective mutualists
Source: Ecol Evol. 2014 Sep 18;4(19):3841–50. doi: 10.1002/ece3.1151 (PMC4301050; doi:10.1002/ece3.1151)
Supplement: Supplementary file 1 — Figure S1 Stability of the Host-Enemy equilibrium. Figure S2 Population size of the host (blue) and enemy (red) populations with sets of parameters predicting either neutrally stable (top caption) or oscillating (bottom caption) equilibrium (see Appendix S1). Figure S3 Stability of the Host-Mutualist equilibrium. [file ece30004-3841-sd1.doc]

**Supplementary figures**

Figure S1 – Stability of the Host-Enemy equilibrium. The grey area indicates the parameter space for which the HE equilibrium is stable (see Appendix 1 – the white area does not allow the equilibrium to exist with positive values). The dashed line is the threshold for which the system is oscillating (oscillations occurs above the line).

Figure S2 – Population size of the host (blue) and enemy (red) populations with sets of parameters predicting either neutrally stable (top caption) or oscillating (bottom caption) equilibrium (see Appendix 1). The same figure can be obtained with the HM equilibrium (not shown).

Figure S3 – Stability of the Host-Mutualist equilibrium. The grey area indicates the parameter space for which the HM equilibrium is stable (see Appendix 1). The dashed line is the threshold for which the system is oscillating (oscillations occurs below the line). It should be noted that whenever the local equilibrium is unstable, it is oscillatory, as is observed for the HE equilibrium. Decreasing the pathogenic effect (alpha) of the mutualist renders this equilibrium less stable. The condition for which the mutualist has no reduction of its pathogenicity (alpha = 1) yields the exact same equilibrium than HE.
