# Supplementary material for: Dispersal and spatial heterogeneity allow coexistence between enemies and protective mutualists
Source: Ecol Evol. 2014 Sep 18;4(19):3841–50. doi: 10.1002/ece3.1151 (PMC4301050; doi:10.1002/ece3.1151)
Supplement: Appendix S1 — Stability of the two-species equilibria [file ece30004-3841-sd2.doc]

Appendix A1 - Stability of the two-species equilibria

In this appendix, we derive the criteria for the local stability of the host–enemy (HE) and host–mutualist (HM) equilibria. Their respective jacobian matrices are

$J_{HE}=\left(\begin{matrix} -\frac{q\delta_{E}}{b\gamma} & -\frac{\delta_{E}}{\gamma} \\ \gamma r-\frac{q\delta_{E}}{b} & 0 \end{matrix}\right)$ (S.1a)

$J_{HM}=\left(\begin{matrix} -\frac{q\delta_{M}}{\alpha b\gamma} & -\frac{\delta_{M}}{\gamma} \\ \gamma r-\frac{q\delta_{M}}{\alpha b} & 0 \end{matrix}\right)$ (S.1b)

Given that these matrices are triangular, we can investigate stability by looking at the sign of their trace and determinants.

**HE equilibrium**

Solving for the two-species equilibrium population sizes in the absence of mutualist yields

$H^{*}=\frac{\delta_{E}}{b\gamma}$ (S.2a)

$E^{*}=\frac{rb\gamma-q\delta_{E}}{b^{2}\gamma}$ (S.2b)

The non-trivial equilibrium point given by equations S.2a and S.2b exists iff

$q<r\frac{b\gamma}{\delta_{E}}$ (S.3)

that is, if the product of enemy growth ($\gamma b/\delta_{E}$) and host growth (*r / q*) are greater than unity.

The two conditions for a locally stable equilibrium are positive Det(J_HE_) and negative Tr(J_HE_). We find that both are always satisfied if the host – enemy equilibrium exists (inequality S.3) and if the host is limited by density dependence in the absence of the enemy (q>0).

Solving the sign of the imaginary parts of the eigenvalues leads to the condition for an oscillatory approach to the locally stable equilibrium point,

$r>q\left(q+\text{4b}\gamma\right)\frac{\delta_{E}}{4b^{2}\gamma^{2}}$ (S.5)

or, in other words, oscillations are more pronounced in highly productive systems. In addition, although sufficiently high values of enemy growth rate ($\gamma$) are necessary for the existence of an equilibrium point, high gamma also increases the range of parameters for which oscillatory approaches to the equilibrium are observed (Figure S1). Extensive numerical experiments confirmed these analytical findings (Figure S2).

**HM equilibrium**

The population sizes of H and M at the two-species equilibrium are

$H^{*}=\frac{\delta_{M}}{b\alpha\gamma}$ (S.6a)

$M^{*}=\frac{rb\alpha\gamma-q\delta_{M}}{\alpha^{2}b^{2}\gamma}$ (S.6b)

and this equilibrium exists iff

$q<r\frac{\alpha b\gamma}{\delta_{M}}$ (S.7)

ir, if the product of mutualist growth ($\left(b\alpha\gamma\right)/\delta_{M}$) and host growth (r / q) are greater than unity. Applying the same local stability analysis as done for the host-enemy couple, we find that if the point exists and q>0, then it is locally stable.

Solving the sign of the imaginary parts of the eigenvalues lead to the condition for a cyclic approach to the stable point,

$\text{4b}\alpha\gamma\left(rb\alpha\gamma-q\delta_{M}\right)>q^{2}\delta_{M}$ (S.9)

As in S.5, increasing gamma will lead to a greater tendency for oscillations away from the equilibrium point (Figure S3).
